# Left Atrial Strain as a Marker of Supraventricular Arrhythmia Risk in Type 2 Diabetes Mellitus

**DOI:** 10.3390/diseases14020064

**Published:** 2026-02-11

**Authors:** Laura-Cătălina Benchea, Larisa Anghel, Vasile Maciuc, Nicoleta Dubei, Răzvan-Liviu Zanfirescu, Gavril-Silviu Bîrgoan, Mircea Ovanez Balasanian, Radu Andy Sascău, Cristian Stătescu

**Affiliations:** 1 Internal Medicine Department, “Grigore T. Popa” University of Medicine and Pharmacy, 700503 Iași, Romania; benchea.lauracatalina@yahoo.com (L.-C.B.); nicoletadubei@yahoo.com (N.D.); silviubirgoan@gmail.com (G.-S.B.); ovanes718@yahoo.com (M.O.B.); radu.sascau@umfiasi.ro (R.A.S.); cristian.statescu@umfiasi.ro (C.S.); 2 Cardiology Department, Cardiovascular Diseases Institute “Prof. Dr. George I.M. Georgescu”, 700503 Iași, Romania; zanfirescu_razvan-liviu@d.umfiasi.ro; 3 Department of Animal Resources and Technologies, Faculty of Food and Animal Sciences, “Ion Ionescu de la Brad” University of Life Sciences, 700489 Iași, Romania; vmaciuc@uaiasi.ro; 4 Physiology Department, “Grigore T. Popa” University of Medicine and Pharmacy, 700503 Iași, Romania

**Keywords:** type 2 diabetes mellitus, atrial myopathy, left atrial strain, supraventricular arrhythmia

## Abstract

Background/Objectives: To determine whether left atrial (LA) strain by speckle-tracking echocardiography can identify supraventricular arrhythmia risk in patients with type 2 diabetes mellitus (T2DM) without overt structural heart disease. Methods: Prospective, single-center observational cohort study including 107 adults: 57 with T2DM and 50 age-matched controls. Participants underwent clinical assessment and echocardiography at baseline and 12 months. LA reservoir, conduit, and contractile strain (LASr, LAScd, LASct) were measured; left atrial volume indexed (LAVI) and LA stiffness index (LASI) were calculated. The primary endpoint was clinically significant supraventricular arrhythmia at 12 months on 24 h Holter (atrial fibrillation (AF)/atrial flutter (AFL) ≥ 30 s and/or excessive supraventricular ectopy). Predictors were assessed using penalized logistic regression and discrimination by ROC analysis. Results: At baseline and 12 months, T2DM showed impaired LA mechanics versus controls (baseline: LASr 20.1 ± 5.7 vs. 25.8 ± 6.3%, LAScd −11.6 ± 4.2 vs. −15.6 ± 4.9%, LASct −9.9 ± 3.2 vs. −13.1 ± 3.7%; all *p* < 0.001) and higher LASI (0.4 ± 0.2 vs. 0.3 ± 0.1, *p* < 0.001). LAVI was higher in T2DM at 12 months (34.0 ± 7.0 vs. 29.9 ± 6.5 mL/m^2^, *p* = 0.003). Supraventricular arrhythmias occurred in 20/57 patients (35.1%) of the T2DM vs. 1/50 patients (2.0%) of the control group (*p* < 0.001). Arrhythmias were assessed by 24 h Holter monitoring at the 12-month follow-up. In T2DM, LAScd provided the best single-parameter discrimination (AUC 0.692), with an optimal cut-off around −8% (sensitivity 55.6%, specificity 81.8%); a LAScd+left ventricular ejection fraction (LVEF) model improved AUC to 0.772. Conclusions: In this prospective observational cohort, T2DM was associated with subclinical LA dysfunction and a higher burden of supraventricular arrhythmias. LAScd emerged as the most clinically informative LA deformation marker for arrhythmic risk stratification and may support targeted rhythm surveillance in diabetic patients. These findings require external validation in larger, independent multicenter cohorts.

## 1. Introduction

Diabetes mellitus (DM) is a chronic metabolic disease that in 2024 affected 589 million people aged between 20 and 79 years, and it is estimated that by 2050, 853 million people will suffer from this disease [1]. DM is a global health problem and is associated with increased morbidity and mortality. For the patient, the burden of diabetes involves years of life lost (YLLs), years lived with disability (YLDs), and disability-adjusted life-years (DALYs). In 2021, there were 37.8 million total diabetes-related YLLs and 41.4 million YLDs, yielding 79.2 million (67.8–92.5) DALYs due to diabetes. Type 2 diabetes mellitus (T2DM) is responsible for 94.0% of diabetes YLLs, 96.6% of YLDs, and 95.4% of DALYs [2]. Cardiovascular diseases (CVD) are the leading cause of mortality in both type 1 and type 2 diabetes. Beyond the increased mortality in this category of patients, if diabetes is associated with cardiovascular manifestations such as myocardial infarction (MI) or stroke, the mortality rate doubles [3]. Given that cardiovascular disease accounts for much of the excess mortality in diabetes, it is particularly important to consider atrial fibrillation as a major and increasingly recognized cardiovascular complication—one for which disorders of glucose metabolism, including diabetes, represent a key modifiable risk factor.

AF is the most common arrhythmia in adults. The European Society of Cardiology Guidelines for the Management of Atrial Fibrillation, published in 2024, illustrate a multitude of risk factors for the occurrence of AF, both non-modifiable and modifiable. Modifiable risk factors include disorders of glucose metabolism, prediabetes, and DM [4]. Diabetes mellitus is an independent risk factor for AF. Patients with DM have a 30% higher risk of developing AF, and this risk increases by 3% each year [5]. Elevated glycosylated hemoglobin (HbA1c) values, together with longer duration of DM, are associated with an increased risk of developing AF [6]. Studies such as ADVANCE (Action in Diabetes and Vascular Disease: Preterax and Diamicron MR Controlled Evaluation) or ORBIT-AF (Outcomes Registry for Better Informed Treatment of Atrial Fibrillation) have shown that patients who associate diabetes with AF have a higher risk of all-cause mortality, cardiovascular mortality, or hospitalization [7,8].

**Figure 1:**
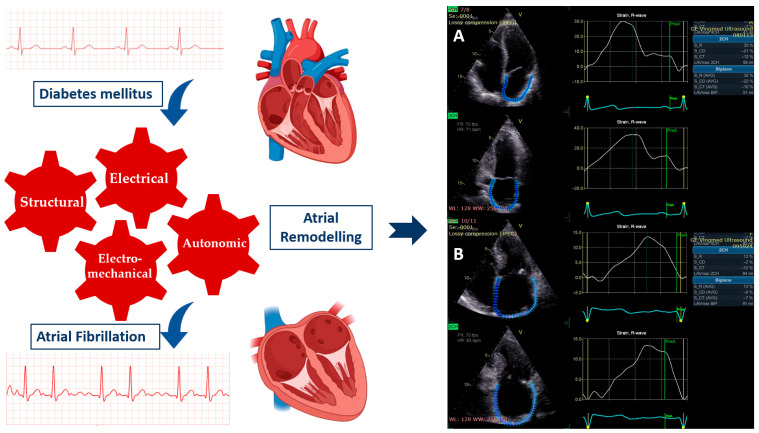
**Left side**. Patients with diabetes mellitus exhibit atrial remodeling at the structural, electrical, electromechanical, and autonomic levels. Atrial myopathy is associated with an increased risk of atrial fibrillation. **Right side**. (**A**) The three functions of the left atrium were evaluated by speckle tracking echocardiography in a patient without diabetes. (**B**) The patient with diabetes has lower values for all three left atrial functions and higher values for left atrial volume.

The substrate for the occurrence of AF in patients with DM is represented by atrial myopathy. This atrial myopathy involves remodeling that occurs at the structural level (dilation and fibrosis), electromechanical level (alteration of excitation-contraction coupling), electrical level (changes in the density of sodium channels and L-type calcium channels), and autonomic level (imbalance between the activity of the sympathetic and parasympathetic nervous systems) (Figure 1) [6]. The pathophysiological mechanisms that determine atrial remodeling include endothelial dysfunction, secretion of pro-inflammatory factors, insulin resistance, fibrinolysis, activation of the renin–angiotensin–aldosterone system (RAAS), acceleration of atherogenesis, and angiogenesis [9].

Beyond structural evaluation of the left atrium (LA), the use of speckle-tracking echocardiography to assess atrial function has shown prognostic value in various clinical settings. American Society of Echocardiography (ASE), in collaboration with European Association of Cardiovascular Imaging (EACVI), recommends tracing the contour of the LA in the apical four-chamber and apical two-chamber views and using special software to determine the three functions of the LA: reservoir (LASr), conduction (LAScd), and contraction (LASct) [10].

In a review published in 2024, the authors showed that reduced LA function in diabetic patients is associated with an increased risk of cardiovascular events such as first AF, congestive heart failure, stroke, transient ischemic attack, myocardial infarction, coronary revascularization, and cardiovascular death [11]. In a retrospective study that included 60 patients (30 adult diabetic patients with documented PAF with 30 age- and sex-matched diabetic patients without paroxysmal AF), Arnautu et al. demonstrated the association between reduced LA strain (LAS) and increased LA stiffness and paroxysmal atrial fibrillation [12]. Sherer J. et al. demonstrated that impaired LAScd is associated with new onset AF, especially when measured two years before AF onset. Inclusion of LAScd in clinical decision-making may lead to individualized assessment of atrial fibrillation risk [13]. Early identification of LA dysfunction can lead to intensified treatment and reduce the risk of AF and its complications, such as stroke.

This original study aimed to evaluate LA strain by speckle-tracking echocardiography as an early marker of supraventricular arrhythmia risk in patients with type 2 diabetes mellitus. We assessed LA reservoir, conduit, and contractile function and examined their association with supraventricular arrhythmias, including AF. By clarifying the relationship between subclinical LA dysfunction and arrhythmic risk, this study aims to support improved risk stratification and earlier preventive strategies in diabetic patients.

Prior work indicates that LA strain is altered in T2DM and linked to atrial myopathy/AF. This study adds prospective, 12-month 24 h Holter-based supraventricular arrhythmia endpoints, focusing on LAScd, a measure of LA passive conduit function during early diastole, and simple, clinically practical risk models in patients without overt structural heart disease. Given the large disease burden of T2DM-related arrhythmias, these findings may have implications for targeted screening and prevention, pending external validation.

## 2. Materials and Methods

### 2.1. Study Design

We conducted a prospective, single-center study at the Cardiovascular Disease Institute ’’Prof. Dr. George I.M. Georgescu’’ in Iași, Romania, designed and reported in accordance with the STROBE (Strengthening the Reporting of Observational Studies and Epidemiology) statement (Table S1). The study aimed to evaluate whether early subclinical signs of LA dysfunction are associated with an increased risk of supraventricular arrhythmias in diabetic patients. Patients were assessed at baseline and after 12 months of follow-up. Speckle-tracking echocardiography was used to assess LA dysfunction, offering a sensitive and quantitative evaluation of myocardial deformation. Recruitment was performed between August 2024 and November 2024, and the 12-month follow-up assessments were completed by November 2025. Participants were consecutively approached from outpatient cardiology clinics at our institute based on predefined inclusion/exclusion criteria. Controls were recruited from routine health evaluations in the same clinical setting and were age-matched; no individual matching was performed for additional variables (e.g., sex or hypertension), although baseline distributions were comparable between groups (Table 1). The study was reviewed and approved by the Ethics Committee of the ’’Grigore T. Popa’’ University of Medicine and Pharmacy in Iași (approval number 464/2024, dated 8 July 2024). All participants gave their written informed consent before entering the study.

### 2.2. Patient Selection and Data Acquisition

This study enrolled 107 adult participants with or without T2DM to evaluate cardiac function using clinical, biochemical, electrocardiographic, and echocardiographic parameters. Data collection was conducted following predefined inclusion and exclusion criteria.

The diabetic group included 57 adult patients (aged > 28 years) with a confirmed diagnosis of T2DM, established in accordance with the 2023 European Society of Cardiology (ESC) guidelines for cardiovascular care in patients with diabetes [14]. The diagnosis of diabetes mellitus was made based on at least one of the following criteria: fasting plasma glucose ≥ 7.0 mmol/L (126 mg/dL), HbA1c ≥ 48 mmol/mol (6.5%), or a 2 h plasma glucose ≥ 11.1 mmol/L (200 mg/dL) following oral glucose loading in the presence of classic hyperglycemic symptoms.

Patients were excluded if they had a history of structural or ischemic heart disease (including valvular heart diseases, cardiomyopathies, congenital heart anomalies, and coronary artery disease), impaired left ventricular systolic function, implanted cardiac devices, significant arrhythmias, chronic pulmonary disease, end-stage renal disease, pregnancy, lactation, or suboptimal echocardiographic image quality.

The control cohort consisted of 50 non-diabetic individuals matched by age, identified through routine health evaluations. The same exclusion criteria applied to the diabetic group were enforced for controls.

Demographic and clinical data were collected, including age, sex, weight, height, body mass index (BMI), body surface area (BSA), calculated using the Du Bois formula, smoking status, alcohol consumption, duration of diabetes, and use of antidiabetic medication. Blood pressure (BP) was measured in accordance with ESC hypertension recommendations [15].

A series of biochemical parameters was determined from fasting morning blood: N-terminal pro-B-type natriuretic peptide (NT-proBNP), plasma glucose, HbA1c, estimated creatinine clearance (mL/min/1.73 m^2^), total cholesterol, low-density lipoprotein (LDL), high-density lipoprotein (HDL), and triglycerides.

A resting electrocardiogram (ECG) was obtained in all participants at baseline and at the 12-month follow-up; additionally, a 24 h Holter ECG monitoring was performed at 12 months to detect supraventricular arrhythmias. Baseline Holter monitoring was not routinely performed. This composite endpoint was chosen to capture clinically meaningful supraventricular rhythm phenotypes reflecting atrial myopathy. AF/AFL episodes lasting ≥30 s are commonly used as clinically relevant arrhythmic events. In addition, excessive supraventricular ectopic activity—often defined as ≥30 PACs/h (≥720 PACs/24 h) and/or runs of ≥20 PACs—has been used in prior literature and has been associated with an increased risk of incident AF and stroke, supporting its clinical plausibility as an early supraventricular marker.

All patients were evaluated by two-dimensional echocardiography and Doppler echocardiography according to the recommendations of the ASE and EACVI guidelines in force [16,17]. We used 2D-echo acquisition (General Electric Healthcare, Vivid E95), analyzed by EchoPAC software (GE EchoPAC PC version 204; GE Vingmed Ultrasound, Horten, Norway). All echocardiographic acquisitions were performed on the same ultrasound system and analyzed offline using the same vendor software following a standardized protocol. Analyses were performed by trained readers blinded to clinical outcomes. LA structure was evaluated using the anteroposterior diameter from the parasternal long-axis view, and LA volume indexed. LA myocardial deformation was assessed using speckle-tracking echocardiography. LA strain value was obtained from non-foreshortened apical four-chamber and two-chamber views using a dedicated LA strain software. All three mechanical phases were evaluated: LASr (in LV systole), LAScd (pre-A-wave diastole), and LASct (late diastole) [10]. In addition, LA stiffness index (LASI) was calculated by the ratio between transmitral flow/annular velocity (E/E’) and LASr. The normal values for LASI according to age are as follows: 0.22 in 20–40 years of age, 0.42 in 40–60 years, and 0.55 in ≥60 years [18]. The Left Atrial Filling Index (LAFI) is an echocardiographic measure assessing LV diastolic function and LV filling pressure. LAFI was calculated as the ratio between the mitral early-diastolic inflow peak velocity (E) and LA reservoir strain [19]. Given the limited number of supraventricular arrhythmia events (*n* = 21 overall; *n* = 20 within T2DM), multivariable modeling was intentionally restricted to minimize overfitting. In the overall cohort, we used Firth penalized logistic regression to reduce small-sample bias and separation; predictor selection prioritized clinical plausibility and model simplicity. In the T2DM subgroup, multivariable models were limited to a maximum of two predictors (LAScd and LVEF), and estimates are interpreted as directional associations.

### 2.3. Statistical Analysis

Statistical analyses were performed using IBM SPSS Statistics, version 23 (SPSS Inc., Chicago, IL, USA). Continuous variables were assessed for distribution (visual inspection and Shapiro–Wilk test) and are reported as mean ± SD for approximately normal data or median (IQR) for non-normal data; categorical variables are presented as *n* (%). Between-group comparisons (T2DM vs. controls) at baseline and at 12 months were conducted using the Mann–Whitney U test for continuous variables and the χ^2^ test for categorical variables, as appropriate. Within-group changes from baseline to 12 months were evaluated using paired t-test. Associations between echocardiographic parameters (e.g., LAScd and LAVI) were assessed using Pearson or Spearman correlation and illustrated with linear regression fits. Key echocardiographic differences are presented with effect sizes and 95% CI where applicable, alongside *p*-values, to facilitate clinical interpretation. The primary endpoint—clinically significant supraventricular arrhythmia at 12 months—was analyzed using logistic regression; given sparse events and potential separation, Firth penalized logistic regression was used for univariate and multivariable modeling, with results expressed as odds ratios (OR) with 95% CI. Discriminatory performance of LA strain parameters and the final multivariable model was evaluated using ROC curves with AUC (95% CI), and optimal thresholds were derived using Youden’s index, reporting sensitivity and specificity. All tests were two-sided, and *p* < 0.05 was considered statistically significant.

## 3. Results

### 3.1. Study Population and Baseline Characteristics

A total of 107 participants were included: 57 with T2DM and 50 controls. All included participants completed baseline and 12-month assessments; therefore, analyses were performed as complete-case. Baseline demographic characteristics were comparable between groups (age 63.3 ± 8.5 vs. 61.1 ± 14.1 years, *p* = 0.327; female sex 50.9% vs. 44.0%, *p* = 0.477). Traditional cardiovascular risk factors were broadly similar, including hypertension (78.9% vs. 76.0%, *p* = 0.715) and smoking (24.6% vs. 18.0%, *p* = 0.410). Dyslipidemia was numerically more frequent in T2DM at baseline (89.5% vs. 80.0%, *p* = 0.170) and became significantly more prevalent at 12 months (91.2% vs. 72.0%, *p* = 0.009) (Table 1).

### 3.2. Echocardiographic Findings and LA Mechanics

Left ventricular (LV) systolic performance was preserved in both groups, without significant between-group differences in conventional LV indices. In contrast, LA remodeling and dysfunction were more pronounced in T2DM. LAVI was higher in T2DM at baseline (32.3 ± 7.3 vs. 29.6 ± 7.5 mL/m^2^, *p* = 0.061) and significantly higher at 12 months (34.0 ± 7.0 vs. 29.9 ± 6.5 mL/m^2^, *p* = 0.003).

Speckle-tracking analysis showed consistently impaired LA mechanical function in T2DM, affecting all three phases:

- reservoir function (LASr) was reduced at baseline (20.1 ± 5.7 vs. 25.8 ± 6.3%, mean difference −5.7%, 95% CI −8.0 to −3.4; *p* < 0.001) and remained reduced at 12 months (19.7 ± 6.0 vs. 26.3 ± 6.6%, mean difference −6.6%, 95% CI −9.0 to −4.2; *p* < 0.001).
- conduit function (LAScd) was less negative in T2DM at baseline (−11.6 ± 4.2 vs. −15.6 ± 4.9%, mean difference +4.0%, 95% CI +2.2 to +5.8; *p* < 0.001) and at 12 months (−10.4 ± 3.9 vs. −15.6 ± 4.8%, mean difference +5.2%, 95% CI +3.5 to +6.9; *p* < 0.001), consistent with reduced conduit deformation magnitude.
- contractile function (LASct) was also less negative in T2DM at baseline (−9.9 ± 3.2 vs. −13.1 ± 3.7%, *p* < 0.001) and at 12 months (−9.3 ± 3.0 vs. −13.5 ± 3.2%, *p* < 0.001).

Indices reflecting diastolic/atrial functional burden were higher in T2DM. LASI was increased at baseline (0.4 ± 0.2 vs. 0.3 ± 0.1, mean difference +0.10, 95% CI +0.04 to +0.16; *p* < 0.001) and at 12 months (0.5 ± 0.3 vs. 0.3 ± 0.1, mean difference +0.20, 95% CI +0.12 to +0.28; *p* < 0.001). The LA filling index was also higher in T2DM at baseline (3.8 ± 1.2 vs. 3.1 ± 0.9, *p* < 0.001) and at 12 months (4.0 ± 1.4 vs. 3.0 ± 0.8, *p* < 0.001) (Table 1).

### 3.3. Supraventricular Arrhythmia Endpoint at 12 Months

At 12 months (Holter monitoring), supraventricular arrhythmias were substantially more frequent in T2DM compared with controls: 20/57 (35.1%) vs. 1/50 (2.0%), *p* < 0.001. The predominant phenotype in T2DM was excessive supraventricular ectopy (ESSV) (18/57, 31.6%), whereas AF was observed in 2/57 (3.5%); atrial flutter was not detected (Table 2).

### 3.4. Association Between LAScd and LA Size

**Figure 2:**
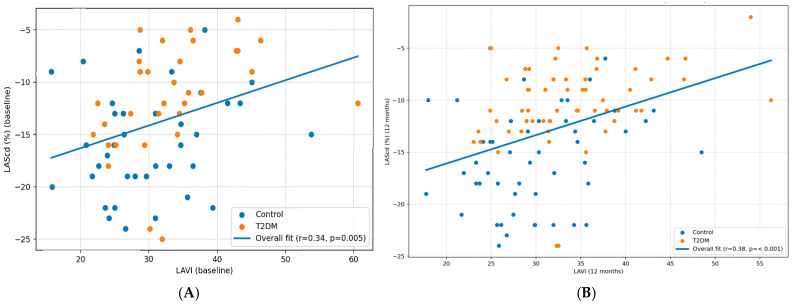
Association between baseline left atrial conduit strain (LAScd) and left atrial volume index (LAVI). (**A**) Scatter plot of baseline LAScd (%) versus baseline LAVI (mL/m^2^) in controls (blue) and patients with type 2 diabetes mellitus (orange). (**B**) Scatter plot of 12 months LAScd (%) versus 12 months LAVI (mL/m^2^) in controls (blue) and patients with type 2 diabetes mellitus (orange).

LAScd correlated positively with LA size (LAVI), indicating that increasing LA volume was associated with less negative LAScd values (i.e., reduced conduit deformation magnitude). The relationship was significant at baseline (r = 0.34 with 95% CI) and strengthened at 12 months (r = 0.38 with 95% CI) (Figure 2). Given the limited number of events, multivariable estimates in the T2DM subgroup should be viewed as hypothesis-generating rather than definitive.

### 3.5. Predictors of Supraventricular Arrhythmia

A total of 21 supraventricular arrhythmia events occurred (20 in T2DM and 1 in controls). In the overall cohort, Firth penalized logistic regression showed that T2DM status was associated with higher odds of supraventricular arrhythmia (OR 31.37, 95% CI 1.67–590.70; *p* = 0.021). Among clinical covariates, smoking was associated with increased risk (OR 4.67, 95% CI 1.06–20.62; *p* = 0.042). LA strain parameters were also related to arrhythmic risk: higher LASr was protective (OR 0.86 per 1% increase, 95% CI 0.76–0.98; *p* = 0.029), whereas less negative LAScd (OR 1.25 per 1% increase, 95% CI 1.02–1.54; *p* = 0.035) and LASct (OR 1.32 per 1% increase, 95% CI 1.02–1.70; *p* = 0.033) were associated with higher risk.

Within the T2DM subgroup, the final multivariable model retained LAScd and LVEF, showing directional associations (LAScd: OR 1.17, 95% CI 0.95–1.43; *p* = 0.138; LVEF: OR 0.91, 95% CI 0.78–1.05; *p* = 0.202).

**Figure 3:**
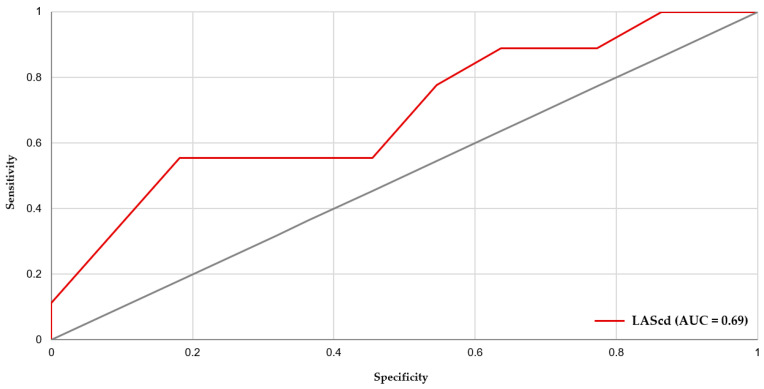
ROC curve of baseline LAScd for predicting 12-month supraventricular arrhythmias in patients with T2DM. T2DM, type 2 diabetes mellitus; LAScd, left atrial strain at conduit.

On ROC analysis in T2DM, LAScd demonstrated the best single-parameter discrimination for supraventricular arrhythmia (AUC 0.692, 95% CI 0.464–0.896), with an optimal threshold of −8% (higher risk if LAScd ≥ −8%), yielding 55.6% sensitivity and 81.8% specificity (Figure 3). In the overall cohort, Firth penalized logistic regression demonstrated that, beyond T2DM status and smoking, multiple LA strain components were consistently related to arrhythmic risk (LASr protective; LAScd and LASct associated with higher risk). Accordingly, ROC analysis in the T2DM subgroup was kept hypothesis-driven and focused on LAScd (the primary conduit-related marker). The multivariable model (LAScd + LVEF) improved discrimination (AUC 0.772, 95% CI 0.566–0.934); using a predicted-probability cutoff of 0.328 provided 88.9% sensitivity and 72.7% specificity. Arrhythmias were assessed by a single 24 h Holter at 12 months; therefore, paroxysmal AF and low-burden supraventricular arrhythmias may have been under-detected. Such misclassification would be expected to attenuate observed associations and may partly explain the moderate AUC values. Future studies using repeated or prolonged monitoring are warranted.

The discriminatory performance of LAScd in T2DM was moderate (AUC 0.692), with modest sensitivity at the ROC-derived cut-off (55.6% at −8%). Accordingly, LAScd should not be interpreted as a stand-alone diagnostic test or a population screening tool to rule out supraventricular arrhythmias. Rather, these findings support LAScd as a risk-enrichment marker that may help identify a subset of T2DM patients who could benefit from more intensive rhythm surveillance (e.g., longer ambulatory monitoring) and closer risk-factor optimization, pending external validation.

## 4. Discussion

Even in the absence of overt structural heart disease and with preserved conventional LV systolic function, T2DM was associated with a pronounced reduction in LA strain across all phases. This pattern is compatible with diabetic atrial cardiomyopathy, in which metabolic and inflammatory pathways promote interstitial remodeling, impaired relaxation, microvascular dysfunction, and altered electromechanical coupling. Importantly, LA strain may capture early dysfunction before substantial chamber dilation becomes evident, which may explain why strain abnormalities were already present at baseline, while differences in LAVI were modest and became clearly significant only at 12 months. In this prospective single-center study, we evaluated LA mechanics by speckle-tracking echocardiography as an early marker of supraventricular arrhythmia risk in patients with type 2 diabetes mellitus. The key findings are: (i) patients with T2DM had significant impairment of LA reservoir, conduit, and contractile function compared with non-diabetic controls at baseline and after 12 months; (ii) clinically significant supraventricular arrhythmia detected by Holter at follow-up was more frequent in the T2DM group; (iii) among LA strain components, conduit strain showed the most consistent association with arrhythmic risk and the best discrimination in ROC analyses; and (iv) LAScd correlated with LA remodeling as reflected by LAVI at both time points, supporting a link between structural enlargement and functional deterioration.

These findings are consistent with the concept of a diabetic atrial cardiomyopathy phenotype, in which atrial dysfunction may be detectable before overt chamber enlargement. This may explain why strain abnormalities were already present at baseline, while differences in LAVI were modest and became clearly significant only at 12 months. Prior studies similarly reported impaired LA strain in T2DM even when conventional LA size indices were still within normal ranges, supporting the use of functional assessment for early detection of subclinical atrial involvement [20].

Among the three LA functional phases, conduit function reflects passive emptying and the interaction between LA compliance and LV early diastolic suction. The finding that LAScd was the strongest discriminator for supraventricular arrhythmia in T2DM suggests that early impairment of passive LA mechanics may represent a key step in arrhythmogenic substrate development. Clinically, “less negative” LAScd values indicate reduced conduit deformation magnitude—compatible with increased stiffness and impaired LA–LV coupling—conditions that may favor atrial stretch, ectopy, and reentry susceptibility. In parallel, the higher LASI and LA filling index in T2DM support the presence of increased diastolic/atrial functional burden; prior work suggests these composite indices may reflect elevated filling pressures and reduced compliance and may add incremental information beyond conventional Doppler metrics [21,22].

The observed correlations between LAScd and LAVI at baseline and 12 months reinforce that functional impairment and chamber remodeling may progress together. Although causality cannot be inferred, larger LA size was associated with worse conduit deformation magnitude, consistent with progressive remodeling. This aligns with prior data showing that higher LAVI in T2DM is associated with lower LA strain values and that LA enlargement and strain impairment may carry prognostic implications, particularly in higher-risk phenotypes [23,24]. However, limited data have specifically examined the longitudinal relationship between LA conduit strain and LA size/volume indices together with systematically assessed Holter-detected supraventricular endpoints in T2DM without overt structural heart disease.

Although LA stiffness index and LA filling index were increased in T2DM, E/E′ itself was not significantly different between groups. This is plausible in a cohort selected to have preserved LV systolic function and no overt ischemic/structural disease, where diastolic impairment may be early and E/E′ can remain within conventionally normal limits. Moreover, E/E′ is influenced by loading conditions and annular velocities and may be less sensitive in preserved EF. In this context, composite atrial markers that integrate transmitral flow with LA deformation (e.g., LASI, LA filling index) may capture a higher atrial functional burden even when E/E′ does not differ.

Previous work has linked reduced LA strain and increased LA stiffness to atrial fibrillation and cardiovascular events in diabetic populations. The present study extends these observations by focusing on a broader supraventricular arrhythmia endpoint detected systematically by Holter at follow-up and by providing longitudinal assessment of LA mechanics and remodeling. If externally validated, LAScd could be integrated as an echocardiographic risk-enrichment marker to complement clinical profiling and biomarkers (e.g., natriuretic peptides) and to guide targeted rhythm surveillance in T2DM—prioritizing prolonged monitoring in higher-risk phenotypes rather than broad screening of all patients.

## 5. Study Limitations

This study has several limitations. First, the sample size was modest and derived from a single center, limiting generalizability and increasing the risk of model overfitting. Second, supraventricular arrhythmias were assessed using 24 h Holter monitoring at 12 months, which may underestimate intermittent or low-burden arrhythmias compared with longer monitoring strategies. Third, the ROC-derived cut-offs and predictive values are dependent on event prevalence in this cohort and should not be directly extrapolated to other populations. Longitudinal blood pressure and glycemic control (HbA1c) during follow-up were not available in a systematic manner for all participants and were therefore not included as time-varying covariates. Residual confounding related to follow-up BP and glycemic control cannot be excluded and should be addressed in future studies. We did not systematically record changes in antidiabetic or cardiovascular medications during follow-up, which may influence LA mechanics and arrhythmia burden.

## 6. Conclusions

In this prospective observational cohort study, type 2 diabetes mellitus was associated with early and persistent LA mechanical dysfunction, consistent with a diabetic atrial myopathy phenotype even in the absence of overt structural heart disease. LA conduit impairment measured by speckle-tracking echocardiography emerged as the most clinically relevant strain component, linking atrial remodeling and elevated filling burden to subsequent supraventricular arrhythmias. Incorporating LA strain—particularly conduit strain—into routine echocardiographic evaluation may improve arrhythmic risk stratification in diabetic patients and support earlier, targeted rhythm surveillance and preventive management. External validation in larger, independent multicenter cohorts is warranted before clinical implementation.

## Figures and Tables

**Table 1:** Baseline and 12-month clinical and echocardiographic characteristics of patients with type 2 diabetes mellitus and controls, with between-group comparisons.

Variable	Baseline		*p*-Value (Baseline)	Follow-Up (12 Months)		*p*-Value (12 Months)
	Patients with T2DM (*n* = 57)	Patients Without T2DM (*n* = 50)		Patients with T2DM (*n* = 57)	Patients Without T2DM (*n* = 50)	
**Clinical Characteristics**						
**Age (years)**	63.3 ± 8.5	61.1 ± 14.1	0.327	63.5 ± 8.5	61.1 ± 14.0	0.302
**Female, *n* (%)**	29 (50.9)	22 (44.0)	0.477	29 (50.9)	22 (44.0)	0.477
**Smoking, *n* (%)**	14 (24.6)	9 (18.0)	0.410	11 (19.3)	9 (18.0)	0.864
**Alcohol user, *n* (%)**	11 (19.3)	10 (20.0)	0.927	4 (7.0)	8 (16.0)	0.142
**Hypertension, *n* (%)**	45 (78.9)	38 (76.0)	0.715	45 (78.9)	40 (80.0)	0.893
**Dyslipidemia, *n* (%)**	51 (89.5)	40 (80.0)	0.170	52 (91.2)	36 (72.0)	0.009
**Echocardiographic characteristics**						
**LVEDV (mL)**	114.9 ± 32.7	110.3 ± 30.4	0.626	118.1 ± 33.9	111.8 ± 29.4	0.306
**LVESV (mL)**	51.2 ± 17.8	48.3 ± 15.9	0.498	52.9 ± 17.5	49.2 ± 15.4	0.334
**LVEF (%)**	55.9 ± 5.2	56.3 ± 4.4	0.627	55.2 ± 4.7	56.5 ± 4.0	0.145
**E/A**	0.9 ± 0.3	1.0 ± 0.3	<0.001	0.9 ± 0.3	1.0 ± 0.3	0.001
**E/E′ septal**	8.9 ± 2.1	8.6 ± 2.5	0.545	9.8 ± 7.6	8.3 ± 1.9	0.136
**E/E′ lateral**	7.5 ± 2.1	7.5 ± 2.3	0.635	7.7 ± 1.7	7.4 ± 1.9	0.520
**GLS (%)**	−15.9 ± 2.9	−18.3 ± 3.4	<0.001	−15.6 ± 2.9	−18.7 ± 2.2	<0.001
**LAVI (mL/m^2^)**	32.3 ± 7.3	29.6 ± 7.5	0.061	34.0 ± 7.0	29.9 ± 6.5	0.003
**LASr (%)**	20.1 ± 5.7	25.8 ± 6.3	<0.001	19.7 ± 6.0	26.3 ± 6.6	<0.001
**LAScd (%)**	−11.6 ± 4.2	−15.6 ± 4.9	<0.001	−10.4 ± 3.9	−15.6 ± 4.8	<0.001
**LASct (%)**	−9.9 ± 3.2	−13.1 ± 3.7	<0.001	−9.3 ± 3.0	−13.5 ± 3.2	<0.001
**LASI (%)**	0.4 ± 0.2	0.3 ± 0.1	<0.001	0.5 ± 0.3	0.3 ± 0.1	<0.001
**LA filling index**	3.8 ± 1.2	3.1 ± 0.9	<0.001	4.0 ± 1.4	3.0 ± 0.8	<0.001

A, filling due to atrial contraction; E, early diastolic filling of the left ventricle; E’, mitral annulus early diastolic velocity measured using tissue Doppler imaging; GLS, Global Longitudinal Strain; LA, left atrium; LAScd, left atrial strain at conduct; LASct, left atrial strain at contraction; LASI, left atrial stiffness index; LASr, left atrial strain at reservoir; LAVI, Left Atrial Volume Indexed; LVEDV, Left Ventricular End-Diastolic Volume; LVEF, Left Ventricular Ejection Fraction; LVESV, Left Ventricular End-Systolic Volume; *n*, number of patients; T2DM, Type 2 Diabetes Mellitus.

**Table 2:** Twelve-month risk of supraventricular arrhythmias in patients with type 2 diabetes mellitus and controls.

Variable	Patients with T2DM (*n* = 57)	Patients Without T2DM (*n* = 50)	*p*-Value
**Atrial fibrillation, *n* (%)**	2 (3.5)	0 (0.0)	0.497
**Premature atrial contractions, *n* (%)**	18 (31.6)	1 (2.0)	<0.001

*n*, number of patients; T2DM, type 2 diabetes mellitus.

## Data Availability

De-identified study data are available on reasonable request from the corresponding author (larisa.anghel@umfiasi.ro). A justification for its further use should be provided.

## Supplementary Materials

The following supporting information can be downloaded at: https://www.mdpi.com/article/10.3390/diseases14020064/s1, Table S1: STROBE Statement—adapted checklist of items to be included in reports of observational studies.

## Abbreviations

The following abbreviations are used in this manuscript:

LA	Left atrium
T2DM	Type 2 Diabetes mellitus
LASr	Left atrial strain at reservoir
LAScd	Left atrial strain at conduct
LASct	Left atrial strain at contraction
LAVI	Left atrial volume indexed
LASI	Left atrial stiffness indexed
AF	Atrial fibrillation
AFL	Atrial flutter
AUC	Area under the curve
LVEF	Left ventricular ejection fraction
DM	Diabetes mellitus
YLLs	Years of life lost
YLDs	Years lived with disability
DALYs	Disability-adjusted life-years
CVD	Cardiovascular Diseases
MI	Myocardial infarction
HbA1c	Glycosylated hemoglobin
RAAS	Renin–angiotensin–aldosterone system
ASE	American Society of Echocardiography
EACVI	European Association of Cardiovascular Imaging
LAS	Left atrial strain
ESC	European Society of Cardiology
BMI	Body mass index
BSA	Body surface area
BP	Blood pressure
NT-proBNP	N-terminal pro-B-type natriuretic peptide
LDL	Low-density lipoprotein
HDL	High-density lipoprotein
ECG	Electrocardiogram
PACs	Premature atrial contractions
LAFI	Left Atrial Filling Index
OR	Odds ratio
ROC	Receiver operating characteristic
LV	Left ventricular
LVEDV	Left ventricular end-diastolic volume
LVESV	Left ventricular end-systolic volume
